# Disrupted Enteric Glial Cell Circadian Oscillation: A Proposed Mechanism Linking CMM-Axis Desynchronization, Gut Jet Lag, and Chronic Functional Constipation

**DOI:** 10.3390/cells15211926

**Published:** 2026-10-23

**Authors:** Yihan Xiong, Lanlan Wang, Yanxi Guo, Yating Lou, Zenghui Yue, Rong Hu

**Affiliations:** College of Acupuncture-Moxibustion, Tuina and Rehabilitation, Hunan University of Chinese Medicine, Changsha 410208, China; 202303010423@stu.hnucm.edu.cn (Y.X.); 20252140@stu.hnucm.edu.cn (L.W.); 202630756@stu.hnucm.edu.cn (Y.G.); 20252044@stu.hnucm.edu.cn (Y.L.)

**Keywords:** enteric glial cells, circadian clock, gut jet lag, chrono-microbiota–motility axis, chronic functional constipation, temporal disruption

## Abstract

**Highlights:**

**What are the main findings?**

- Within the multi-cellular gut circadian network, this review proposes enteric glial cells as one potential interstitial circadian relay linking host timing signals, gut microbial rhythms, and colonic motility within the Chrono-Microbiota–Motility axis.
- Disrupted enteric glial rhythms may contribute to chronic functional constipation by impairing host–microbiota synchronization and altering the daily pattern of colonic motility.

**What are the implications of the main findings?**

- Enteric glial clocks warrant investigation as potential therapeutic targets in chronic functional constipation associated with circadian disruption.
- Circadian disruption may be relevant to constipation risk stratification, particularly among shift workers and individuals with chronic sleep disturbance.

**Abstract:**

Chronic functional constipation is highly prevalent and frequently recurrent, and gut jet lag triggered by circadian-rhythm disruption represents an important modern-lifestyle-associated risk factor. Although the Chrono-Microbiota–Motility (CMM)-axis conceptual framework for circadian-disruption-related functional constipation has been published, existing work mainly describes macroscopic microbiota–motility crosstalk and largely overlooks enteric glial cells (EGCs) as secondary peripheral circadian relays within the gut wall. Drawing on evidence from population-based epidemiology, human colonic specimens, multi-omics studies, and animal models of circadian disruption, this review delineates the pathological mechanisms by which disrupted circadian oscillations of enteric glial cells mediate hierarchical desynchronization of the CMM axis and contribute to gut jet lag. It further outlines a progressive pathological cascade from external circadian perturbations and enteric glial cell clock disruption to host–microbiota temporal desynchronization and subsequent collapse of the colonic motility network, discusses the potential implications of this mechanism for clinical risk stratification in chronic functional constipation, and provides a theoretical basis for future basic and translational research in gut chronobiology. It should be emphasised that the integrated mechanistic framework presented herein constitutes a testable hypothesis largely built upon pre-clinical animal observations and indirect human tissue evidence, rather than an established pathophysiological reality. This framework highlights one potential interstitial branch within the complex multi-cellular gut circadian regulatory network.

## 1. Introduction

Chronic functional constipation (CFC) is a common functional gastrointestinal disorder characterized by infrequent bowel movements, hard stools, straining, and a sensation of incomplete evacuation. Approximately 10% of adults worldwide are chronically affected by CFC. Symptoms frequently recur, conventional treatments often provide suboptimal relief, and the associated disease burden remains substantial [1]. Previous discussions of its etiology have largely focused on insufficient dietary fiber intake, physical inactivity, and poor bowel habits [2,3]. By contrast, circadian rhythm disruption, an increasingly prevalent risk factor associated with modern lifestyles, has long received insufficient attention. Shift work and sleep deprivation are becoming increasingly common among occupational populations. Both can shift daily cues and behaviors, including light exposure, eating, activity, and rest, away from their habitual timing, thereby disrupting circadian rhythms. The gut is particularly sensitive to such temporal misalignment [3,4]. Chang and Peng [5] analyzed gastrointestinal symptom data from shift workers and fixed-day workers and found that constipation was reported significantly more frequently among shift workers. In addition, several population-based cohort studies have shown that shift work and sleep deprivation are associated with a 2.1- to 2.8-fold higher risk of CFC, with a dose–response relationship between the degree of circadian phase disruption and constipation severity [6]. Adults with sleep disorders also have a 65% higher risk of constipation than those without sleep disorders [7]. Together, these findings point to circadian rhythm disruption as a modifiable risk factor for constipation.

In recent years, the relationship between circadian rhythms and gut health has attracted increasing attention, although most studies have remained at a relatively broad level. Li et al. [8] introduced the concept of “gut jet lag,” defining desynchronization between the host circadian clock and microbial rhythms as its core mechanism. On this basis, Li et al. proposed the theoretical framework of the Chrono-Microbiota–Motility (CMM) axis, bringing the etiology of constipation from a predominantly metabolic perspective into the context of biological rhythms for the first time and opening a new direction for research. However, this framework overlooked the central role of enteric glial cells (EGCs), which act as secondary peripheral circadian clocks in the gut and serve as key relays for temporal signals. Focusing on the relationship between circadian rhythms of the gut microbiota and nocturnal hyperglycemia in type 2 diabetes mellitus, Ying et al. systematically reviewed microbial metabolites, the intestinal barrier, and neuroendocrine pathways, and proposed intervention strategies such as time-restricted eating [9]. Building on these advances, the intrinsic temporal regulatory mechanisms of the gut warrant further attention.

The gut harbors a relatively autonomous peripheral clock, and the enteric nervous system and its glial cells also exhibit rhythmic functional properties. EGCs lie at the interface between microbial signaling and enteric neural control of gut motility. Disruption of their intrinsic rhythms may translate external circadian desynchronization into local temporal disorganization within the gut, thereby driving the sequential uncoupling of the microbiota, interstitial cell network, and gut motility [10,11]. Although previous reviews have described the links among circadian rhythms, the gut microbiota, and intestinal motility, most have treated the gut as a homogeneous entity rather than focusing on cells within the intestinal wall, particularly the pivotal role of EGCs in relaying temporal signals. A systematic cellular-level account of how the CMM axis becomes desynchronized is therefore still lacking. Much of the existing literature has also viewed the gut primarily as an effector organ that passively receives signals from the central clock and the microbiota. The cell types within the intestinal wall that constitute secondary peripheral clocks, and the molecular processes through which rhythmic signals are transmitted sequentially across these cell populations, have yet to be systematically characterized.

Gut circadian physiology represents an orchestrated multi-cellular network process coordinated by the SCN central clock, enteric neurons, enteric glial cells, intestinal epithelial cells, interstitial pacemaker cells and immune cell populations [3]. This review focuses on one testable interstitial branch within this larger multi-cellular network: the potential contributions of disrupted EGC circadian function toward CMM-axis desynchronization and gut jet lag, without arguing that EGCs act as the single dominant or primary regulatory cell type.

Specifically, this article has two main aims. First, it elucidates the molecular basis by which EGCs can act as one interstitial relay component linking central circadian signals, the gut microbiota, and colonic motility to maintain temporal homeostasis of the CMM axis under physiological conditions. Second, it delineates step-by-step how circadian perturbation, via EGC dysfunction, drives hierarchical CMM-axis desynchronization, gut jet lag and subsequent chronic functional constipation. We further discuss the implications of this hypothesis for future clinical risk-stratification and basic research on chronic functional constipation.

## 2. Physiological Basis: Enteric Glial Cell Circadian Rhythms as a Core Relay for CMM Axis Homeostasis

The physiological homeostasis of the CMM axis depends on multilayer temporal synchronization among the central clock, the gut microbiota, and colonic motility, with EGCs positioned at the convergence of these signals. On the one hand, EGCs receive entrainment cues from the suprachiasmatic nucleus (SCN) and feeding rhythms to maintain their peripheral circadian clock. On the other hand, they relay temporal information to the gut microbiota and the gut wall motor network, thereby coordinating the host clock, microbial metabolism, and colonic motility across the 24 h cycle. Of note, such EGC-mediated temporal signalling operates within a broader multi-cellular gut circadian network, where enteric neurons, epithelial cells and interstitial cell populations also exert independent temporal regulatory functions.

### 2.1. Entrainment of the EGC Peripheral Clock by the Central SCN

In mammals, circadian rhythms are not governed by a single clock but by a hierarchical network of central and peripheral oscillators [12]. The central clock resides in the SCN of the hypothalamus, where it receives external time cues such as light and conveys phase information to peripheral clocks throughout the body. These peripheral clocks are distributed across organs and tissues, where they respond to central entrainment signals while maintaining local autonomous oscillations [12,13]. Within this hierarchical network, the gut is not merely a passive recipient of SCN-derived temporal signals. Multiple cell types in the intestinal wall possess cell-autonomous circadian oscillators and together form a secondary peripheral clock network in the gut [14,15]. In response to light, the SCN encodes temporal information into neural signals that are relayed through nuclei such as the hypothalamic paraventricular nucleus and then transmitted to the intestinal wall via autonomic pathways, including the dorsal motor nucleus of the vagus (parasympathetic) and the intermediolateral cell column of the spinal cord (sympathetic) [16,17]. The SCN also indirectly entrains the intestinal clock through neuroendocrine routes, including hypothalamic–gut pathways, with humoral signals such as glucocorticoids and melatonin [16]. Regular feeding provides an additional entrainment pathway that is partly independent of the central clock, allowing feeding-related signals to reset peripheral clocks in the gut without altering the phase of the SCN [18].

At the tissue level, the stomach and colon exhibit distinct peripheral clock properties. The phase of Bmal1 in the stomach and colon differs from that in the SCN by no more than approximately 3 h, whereas the phase difference for Per2 can reach 10 h. This indicates that, although the intestinal clock is entrained by the central clock, it retains tissue-specific flexibility in phase, and individual clock genes are not necessarily locked to the same phase [19]. At the cellular level, Magaña et al. [14] recorded oscillations in different cell types within the intestinal muscularis externa and demonstrated that enteric neurons, EGCs, smooth muscle cells, macrophage-like cells, and interstitial cells of Cajal can each sustain autonomous circadian oscillations. In EGCs, these autonomous oscillations are maintained by the canonical mammalian CLOCK/BMAL1–PER/CRY transcriptional–translational feedback loop, in which CLOCK/BMAL1 promotes the transcription of clock genes such as Per and Cry, whereas the subsequent accumulation of PER/CRY proteins suppresses CLOCK/BMAL1 activity, thereby generating oscillations with a period of approximately 24 h [14,20]. Altering the feeding–fasting cycle can also reset the phase of EGC oscillations, further demonstrating that their intrinsic clock not only sustains autonomous rhythmicity but also responds to local metabolic entrainment cues. EGCs therefore possess both cell-autonomous timekeeping capacity and responsiveness to external entrainment signals, allowing them to preserve intrinsic temporal information while adjusting their phase in accordance with feeding rhythms.

### 2.2. EGC–Microbiota Temporal Coupling in CMM Layer 1: Host–Microbiota Synchronization

Studies have shown that the composition, functional activity, and metabolites of the gut microbiota all exhibit circadian rhythmicity. This rhythmicity is reflected not only in 24 h fluctuations in the abundance of different microbial taxa, but also in daily variations in microbial functional gene expression and in metabolites such as short-chain fatty acids (SCFAs) and bile acids [21]. These regular temporal changes coordinate microbial metabolic activity with host feeding, barrier function, and metabolic state, providing an important basis for host–microbiota homeostasis. Unlike the SCN, the gut microbiota does not possess a unified central clock; its circadian fluctuations are shaped primarily by the host circadian clock and the feeding–fasting cycle [21].

EGC-mediated temporal regulation of the microbiota is functionally partitioned across the day–night cycle, with distinct daytime and nighttime peaks [14,22]. During the daytime, EGCs primarily regulate microbial proliferation and metabolic activity. Bile salt hydrolase (BSH) is a key microbial enzyme involved in bile acid metabolism and catalyzes the hydrolysis of conjugated bile acids [23]. Metatranscriptomic sequencing of mouse cecal samples showed that BSH gene expression peaks during the daytime [24], while enzyme activity assays using mouse intestinal samples demonstrated that BSH activity follows a circadian rhythm and is influenced by feeding patterns, indicating an active daytime phase of microbial bile acid metabolism [23,25]. Meanwhile, in mouse EGC-depletion experiments, EGC-mediated regulation of antimicrobial secretion by Paneth cells can alter microbiota composition [22], indicating that Paneth cells and the mucosal microenvironment they shape can serve as a functional interface through which EGCs influence microbial ecology. In a human observational study, Shimizu et al. [26] further reported that shorter sleep duration was associated with reduced secretion of Paneth cell-derived human α-defensin 5 (HD5), together with lower abundances of SCFA-producing bacteria such as Butyricicoccus and reduced fecal SCFA levels. HD5 levels were also positively correlated with the abundance of these bacteria and with SCFA concentrations, suggesting that sleep-related circadian disruption may be accompanied by parallel changes in Paneth cell antimicrobial output and microbial metabolic function. Collectively, these separate lines of indirect experimental and observational evidence support a testable proposed pathway: EGCs may release growth-promoting signals during the daytime that act through Paneth cells and the intestinal epithelial microenvironment to drive a daytime increase in the abundance of butyrate-producing and bile salt-hydrolyzing bacteria, thereby supporting daytime microbial bile-acid metabolism. No direct in vivo circadian-resolved experiments have yet validated this sequential EGC–Paneth–microbiota cascade; this represents a speculative mechanistic chain rather than an experimentally established pathway.

The nighttime phase is characterized primarily by temporal regulation of intestinal epithelial barrier permeability. EGCs are important for maintaining intestinal epithelial barrier homeostasis. Using mouse-derived EGCs and in vitro epithelial co-culture and organoid models, Meir et al. [27] found that glial cell line-derived neurotrophic factor (GDNF) released by EGCs can promote barrier maturation and maintain barrier integrity through epithelial RET signaling. In addition, studies involving transgenic mice and in vitro epithelial co-cultures, together with complementary ex vivo evidence from human colonic biopsy specimens, have shown that EGCs can regulate epithelial tight junctions and barrier permeability by releasing S-nitrosoglutathione (GSNO) [28]. Hunter et al. [29] further showed in mice that intestinal epithelial tight junctions and permeability exhibit HDAC3-dependent circadian rhythms, with permeability being higher at dusk than at dawn. During the night, rhythmic EGC output through mediators such as GDNF and GSNO is coupled to phase-dependent changes in barrier permeability. The interplay between EGC-mediated regulation of barrier function and the intrinsic circadian rhythm of the barrier renders molecular exchange between the host and the microbiota distinctly time-dependent, restricting the exchange of microbial metabolites such as SCFAs and bile acids with host tissues during specific nighttime periods. This temporal gating thereby contributes to maintaining the 24 h rhythmic release of microbial metabolites.

Temporal signaling from the host to the microbiota via EGCs is not unidirectional. Using mouse-derived intestinal organoids and complementary human-derived intestinal epithelial models, Fawad et al. [30] found that microbiota-derived SCFAs can feed back on the intestinal epithelial clock through histone deacetylase inhibition. Furthermore, intestinal epithelial-specific Bmal1 knockout in mice disrupted the circadian rhythmicity of the microbiota and markedly altered microbial metabolites, including branched-chain fatty acids and secondary bile acids [31], thereby validating the interdependence of this bidirectional coupling from the host side. Together, these findings suggest that EGCs may occupy a key relay position within this bidirectional feedback loop and provide a physiological basis for temporal synchronization between the host and the microbiota. However, direct time-resolved evidence demonstrating EGC-dependent host–microbiota synchronization in humans remains lacking.

### 2.3. Temporal Coupling of EGCs with ICCs and PDGFRα^+^ Motility Cells in CMM Layer 2: Temporal Execution in the Gut Wall

EGCs not only relay host-derived temporal information to the microbiota but also directly participate in the regulation of the gut wall motor network [32]. Interstitial cells of Cajal (ICCs) and platelet-derived growth factor receptor α-positive (PDGFRα^+^) cells determine the contractile rhythm of the colon. ICCs generate slow waves and drive smooth muscle activity through gap junctional coupling, whereas PDGFRα^+^ cells mediate purinergic inhibitory input. Together with smooth muscle cells, these two cell types form the smooth muscle cell–ICC–PDGFRα^+^ cell (SIP) syncytium, through which contractile rhythmicity is generated [33,34]. Studies using ex vivo mouse intestinal tissues have shown that although ICCs possess autonomous oscillators, these oscillators cannot be entrained by feeding–fasting cycles, whereas EGC oscillators can be entrained by feeding rhythms [14]. Both EGCs and ICCs express the purinergic receptor P2Y4 in guinea-pig gastrointestinal tissue and can communicate through intracellular Ca^2+^ waves, providing a cellular basis for direct functional communication between the two cell types [35]. EGCs can undergo phase resetting in response to feeding signals, whereas ICCs are insensitive to feeding–fasting entrainment cues. This difference suggests that feeding-related temporal signals need to pass through entrainment-responsive intermediary cells before reaching motor effector cells such as ICCs, providing a temporal basis for the proposed phase-relay function of EGCs.

Sánchez et al. [36] observed diurnal variation in plasma 5-HT levels in rats. Jin et al. [37] further demonstrated in diabetic mouse models that 5-HT can enhance ICC pacemaker activity and promote colonic motility through 5-HT_2_B receptors on ICCs, consistent with the activity-phase peak in defecation observed in 24 h human manometric studies [38,39]. Together, these findings indicate a functional correspondence between circadian variation in 5-HT, ICC pacemaking, and enhanced colonic motility during the active phase, providing an important excitatory regulatory basis for circadian variation in the gut wall motor network. By contrast, colonic motility remains relatively low during the resting phase, and PDGFRα^+^ cell-mediated purinergic inhibition may contribute to this low-motility state. Lee et al. [40] showed in human colonic specimens that PDGFRα^+^ cell-mediated purinergic neurotransmission can inhibit colonic contractions, providing a cellular basis for motility suppression during the resting phase.

EGC regulation of motor cells is not unidirectional; the microbiota also feeds signals back to EGCs and the gut wall. In mouse experiments, the gut microbiota can continuously activate mucosal EGCs via TLR4 and promote the release of ciliary neurotrophic factor [41], indicating that microbial signals can directly alter the state of EGCs. In mouse colonic preparations, microbiota-derived SCFAs can activate myenteric neurons through FFAR2/3 and slow colonic transit [42]. In mice subjected to chronic jet lag, serum bile acid rhythms were lost and TGR5 (Gpbar1) signaling in the smooth muscle layer was remodeled [43], corroborating the finding by Alemi et al. [44] that bile acids promote colonic motility through TGR5 in mice. Taken together, EGCs receive entrainment signals from the central clock and feeding rhythms while also coupling bidirectionally with the gut microbiota and the SIP motor syncytium, thereby forming a cellular relay node that maintains the physiological homeostasis of the CMM axis (Figure 1).

Microbial-metabolite-sensing receptors including FFAR2/FFAR3 (short-chain fatty acid receptors) and TGR5 (bile-acid-sensing receptor) are expressed on both enteric neurons and subsets of EGC populations [45,46]. The transcript and protein levels of these metabolite-sensing receptors themselves undergo circadian modulation, which determines the sensitivity of enteric neural–glial networks to rhythmic microbial metabolite signals across the 24 h cycle. When receptor expression exhibits day–night oscillation, the same concentration of short-chain fatty acids or bile acids can trigger distinct cellular responses at different circadian time points [47]. Through these receptors, microbial metabolites can shape circadian rhythmicity of enteric neurons and EGCs, and further translate microbial metabolic signals into time-dependent output to downstream colonic motility effector cells such as ICCs and PDGFRα^+^ cells [47], ultimately helping to configure diurnal colonic-motility patterns. Notably, such time-dependent receptor-mediated signaling can occur not only via enteric neurons but potentially also through direct activation of subsets of EGCs [45]. At present, temporally resolved receptor-expression profiles specifically obtained from human EGCs remain scarce [48]. Most existing functional observations are derived from rodent models. Whether and how rhythmic receptor expression in human EGCs modulates gut circadian rhythms and colonic motility still represents an important outstanding knowledge gap for future investigation. Of note, the above-described mechanistic cascade is predominantly supported by pre-clinical rodent and in vitro experimental data; comparable temporally resolved human intestinal tissue evidence remains very limited.

### 2.4. Brief Overview of Multi-Cellular Orchestration Within Gut Circadian Network

Gut circadian homeostasis is achieved via hierarchical signal propagation and reciprocal feedback across multiple clock-bearing cell populations, rather than being governed by a single dominant cell type. At the top of this network, the SCN provides central master timing signals transmitted through autonomic neuro-output and humoral mediators. These global timing inputs are then distributed to diverse intestinal cell compartments. Enteric neurons act as primary signal-integrating hubs: they receive SCN-derived central timing, local glial mediators, and microbial metabolite inputs, and convert multi-source temporal information into excitatory or inhibitory outputs toward gut effector cells.

EGCs function as interstitial relay nodes in parallel with enteric neurons. They are independently entrainable by feeding-related metabolic cues, receive neuronal inputs, and in turn secrete glial mediators to fine-tune the phase and amplitude of neuronal, epithelial, and interstitial cell activity. This creates a parallel interstitial signalling stream alongside canonical neuronal pathways. Intestinal epithelial cells integrate neuronal–glial signals and microbial metabolite stimuli to set rhythmic barrier permeability and antimicrobial secretion; epithelial clock outputs further feed back to reshape microbial diurnal rhythms. Downstream interstitial pace-maker cells (ICCs and PDGFRα^+^ cells) decode combined neuronal–glial temporal signals and generate time-dependent smooth-muscle contractile responses that produce colonic motility patterns.

Completing this regulatory loop, gut-resident immune cells sense tissue activity and microbial products, exerting time-gated inflammatory feedback that can reset or dampen cellular clock oscillations of neurons and glia. All these compartments continuously calibrate one another’s phase and functional amplitude through bidirectional signalling (Figure 2). Within this complex multi-layered network, the present review specifically focuses on the testable pathophysiological consequences of EGC circadian disruption—a parallel interstitial signalling branch that has not been systematically synthesised in previous CMM-axis-oriented reviews.

## 3. Collapse of Enteric Glial Cell Circadian Rhythms as a Core Pathological Pathway Driving Gut Jet Lag and CMM Axis Desynchronization

### 3.1. External Circadian Perturbations Disrupt the Intrinsic Circadian Oscillations of EGCs (Initiation Stage)

The circadian oscillations of EGCs are cell-autonomous, although their phase is continuously calibrated by the central clock and feeding rhythms [14]. Circadian perturbations, including night shifts, rotating shifts, bright light at night, irregular feeding, and sleep deprivation, cause timing cues such as light exposure, sleep, and food intake to occur at inappropriate times, resulting in misalignment between the central and peripheral clocks. Fishbein et al. described this state as a misalignment of the external light–dark cycle, social behavior, and the timing of sleep and food intake with the endogenous circadian system [49]. Animal studies further show that chronic jet lag shifts the expression phases of most core clock genes in the SCN, whereas constant light disrupts synchrony among SCN neurons [50,51]. Misaligned SCN output can then transmit aberrant timing signals to gastrointestinal peripheral clocks through autonomic and humoral pathways.

At the same time, feeding time itself is an important peripheral zeitgeber for the gut, making it necessary to distinguish between two major pathways. Disruption of the light–dark cycle primarily affects EGCs indirectly through SCN-mediated autonomic and humoral pathways, whereas mistimed feeding can directly reset the peripheral clocks of EGCs and other cells in the intestinal wall, partly independently of the SCN. Cell-level temporal evidence supports a direct response of EGCs to mistimed timing cues. When feeding is shifted to the inactive phase, EGCs, enteric neurons, and other intestinal wall cells undergo phase shifts to varying degrees and transition from a synchronized state to intercellular desynchronization [14]. Similarly, SCN-specific Bmal1 deficiency and simulated shift-work models show desynchronization of peripheral clocks across different regions of the gastrointestinal tract [52], while circadian phase shifts in clock-gene expression have been detected in the colonic smooth muscle layer in a chronic jet lag model [43]. Together, these findings indicate that circadian perturbations can directly induce phase misalignment in EGCs, accompanied by disruption of synchrony among intestinal wall cells. If such misalignment persists, peripheral clocks may further exhibit reduced oscillation amplitude and impaired rhythm stability, allowing transient failure of re-entrainment to gradually develop into persistent temporal instability.

For EGC clock abnormalities to further drive CMM axis desynchronization, a critical step is the transmission of molecular timing information to glial functional outputs. The CLOCK/BMAL1–PER/CRY loop not only sustains the oscillation of clock genes themselves but also regulates cellular metabolism, Ca^2+^ signaling, inflammatory programs, and mediator release through clock-controlled transcription [32]. Relevant mechanistic insights are partially derived from investigations of central astrocytes. In primary astrocytes, extracellular ATP release exhibits a clear circadian rhythm, whereas disruption of core clock-gene function markedly attenuates or abolishes this rhythmic release [53]. These astrocyte-based findings provide analogical support for the concept that glial clock disruption may alter rhythmic mediator output, yet they cannot serve as direct proof for equivalent clock-controlled behaviour in intestinal EGCs.

Recent cell-type-specific studies have further strengthened this link. McKee et al. [54] showed that astrocyte-specific Bmal1 deletion markedly alters cellular processes including endocytosis, lysosome-dependent protein degradation, and autophagy. Disruption of Bmal1 in astrocytes also induces a reactive glial phenotype accompanied by altered expression of inflammation-related molecules [55]. Together, these findings further indicate that the glial cell-autonomous clock not only determines oscillatory states but also contributes to the maintenance of glial function and immune phenotype.

External circadian perturbations do not necessarily progress to persistent collapse of the EGC clock; individual capacity for re-entrainment and circadian recovery may determine whether this process continues. Sellix et al. [56] found that aging markedly alters the re-entrainment of central and peripheral clocks following phase shifts, suggesting that advancing age may reduce the capacity of the circadian system to recover from repeated misalignment. In addition, genetic variation in core clock genes can itself alter intrinsic circadian period and phase characteristics. For example, Patke et al. [57] demonstrated that a functional variant in CRY1 can lengthen the endogenous circadian period and delay sleep phase. Previous intestinal inflammation may also leave a persistent background of glial–immune abnormalities. Grubišić et al. [58] found that EGC-related signaling after acute colitis can sustain abnormalities in barrier function and inflammatory responses, providing experimental evidence that prior intestinal injury may alter subsequent stress responses of EGCs. Thus, the same external circadian perturbations, such as night shifts, sleep deprivation, and mistimed feeding, can lead to markedly different outcomes across individuals. Those with greater capacity for circadian re-entrainment may experience only transient and reversible phase shifts, whereas the presence of susceptibility factors such as aging, clock-gene polymorphisms, and prior intestinal injury may facilitate progression from transient phase misalignment to persistent instability of peripheral circadian rhythms.

### 3.2. EGC Rhythm Disruption Impairs Host–Microbiota Synchrony and Drives Early Gut Jet Lag (First-Layer CMM Desynchronization)

Loss of rhythmic glial-factor secretion by EGCs first compromises their temporal regulation of the gut microbiota. EGCs relay temporal signals to the microbiota through Paneth cells; in a mouse EGC-depletion model, selective depletion of PLP1^+^ EGCs reduces the secretion of functional lysozyme by Paneth cells, accompanied by changes in fecal microbial composition [22]. In a human observational study, Shimizu et al. [26] further found that shorter sleep duration was associated with reduced secretion of Paneth cell-derived HD5, together with lower abundances of SCFA-producing bacteria such as Butyricicoccus and reduced fecal SCFA levels. HD5 levels were positively correlated with the abundance of these bacteria and with fecal SCFA concentrations, further suggesting that circadian-related perturbations can alter microbial composition and metabolic function through Paneth cell antimicrobial output.

Once circadian oscillations of the microbiota are lost, changes in metabolite rhythmicity are among the first features to emerge. Using targeted metabolomics, Heddes et al. [31] found that microbiota-derived SCFAs and bile acids no longer exhibited circadian rhythms in mice lacking an intestinal clock. The circadian rhythm of serum bile acids was likewise completely abolished in mice subjected to chronic jet lag [43]. Following intestinal epithelial-specific Bmal1 knockout, the number of microbial taxa exhibiting circadian oscillations decreased by approximately two-thirds, and multiple previously rhythmic taxa, including Lachnospiraceae and Ruminococcaceae, became arrhythmic [31]. These findings directly demonstrate that disruption of a local host clock is sufficient to cause widespread loss of 24 h oscillations in microbial abundance under experimental rodent conditions. From the perspective of exogenous entrainment, Cui et al. [59] further showed in mice that simply shifting feeding to the rest phase simultaneously altered the diurnal fluctuations of host clock genes, fecal bile acids, and multiple microbial taxa, indicating that mistimed feeding alone is sufficient to disrupt temporal synchrony between host metabolism and the microbiota. Leng et al. [60] provided complementary evidence from an intervention perspective, showing in a chronic jet-lag mouse model that time-restricted feeding restored jejunal metabolic rhythms disrupted by chronic jet lag toward their normal pattern, suggesting that rhythmic output of microbial metabolites can likewise recover, together with partial restoration of the disrupted intestinal clock and metabolic transcriptional rhythms. The same study also included in vitro experiments using mouse-derived jejunal enteroids to investigate epithelial clock-dependent rhythmic processes. However, direct time-resolved human evidence linking EGC-specific circadian disruption to host–microbiota desynchronization remains lacking.

### 3.3. Failure of EGC Temporal Regulation Drives Collapse of Circadian Function in Colonic Motility Cells (Second-Layer CMM Desynchronization, Core Pathogenic Step)

Following the establishment of first-layer temporal desynchronization between the host and the microbiota, the disruption begins to extend further to the motility layer of the intestinal wall. Salgado-Delgado et al. [61] found in rats that feeding during the rest phase not only desynchronizes metabolic and neuronal rhythms but is also accompanied by impaired gastric emptying and gastrointestinal motility, suggesting that persistent temporal misalignment can gradually propagate from the metabolic level to the neural–motility system. At this stage, EGC-mediated circadian regulation of colonic motility breaks down further. High-amplitude propagated contractions (HAPCs) are an important form of propulsive colonic motility. Twenty-four-hour human manometry studies show that HAPCs occur predominantly after morning awakening, are most frequent in the early morning, and are least frequent at night; accordingly, defecation is concentrated in the morning, producing a morning peak in bowel movements [38,39]. This overall pattern of motility indicates that colonic propulsive activity itself has a distinct temporal organization. Under physiological conditions, the temporally patterned functional output of EGCs may form part of the temporal information within the enteric neural network and, together with the intrinsic cellular clocks of neurons and the SIP motility network, maintain a motility pattern characterized by enhancement after morning awakening and meals and attenuation at night [14]. When rhythmic secretion by EGCs is lost and GDNF no longer exhibits circadian phase variation, EGCs transmit temporal signals indirectly to the SIP syncytium, primarily through myenteric neurons serving as an intermediate relay [62]. This represents a proposed multi-step mechanistic model. Upon loss of rhythmic EGC-derived signalling output, myenteric neurons may lose phase-dependent excitatory input, which could further attenuate the daytime peak of ICC Ca^2+^ transients, markedly reducing or even abolishing HAPCs [34,62]. It should be emphasized that HAPC generation is regulated by overlapping neuronal, hormonal, muscular and luminal–microbial inputs [38]. Direct experimental evidence specifically linking EGC clock-oscillation disruption to diminished HAPC amplitude or frequency is still absent. The phase organization of alternating resting and activated states in PDGFRα^+^ cells may also be disrupted, which could flatten global slow-wave rhythms across the day [34]. Consequently, colonic motility no longer retains an intact 24 h temporal structure [34].

Circadian rhythm instability disrupts the temporal pattern of colonic motility. Using 24 h ambulatory manometry in healthy human volunteers, Rao et al. [63] found that colonic motor activity is greatest after morning awakening and meals. In awake rats, luminal SCFAs stimulate enterochromaffin cells (EC cells) to release 5-HT, which subsequently accelerates colonic transit through 5-HT_3_ receptors [64]. The rhythmic secretion of 5-HT, however, depends on GDNF support from EGCs [65]. This pathway is also regulated by the intestinal clock, and the TPH1–5-HT signaling pathway in EC cells exhibits distinct circadian variation in mouse experiments [66]. 5-HT released by EC cells is an important mediator linking luminal stimuli to enteric neural–motility responses [67,68].

Segers et al. [47] found in mice that both fecal SCFA levels and SCFA-induced colonic contractions exhibit circadian rhythms. In an immortalized rat EGC cell line, butyrate and propionate, two major SCFAs, directly alter glial mediator output [69]. Microbiota-derived pro-motility signals in the form of SCFAs are themselves rhythmic and also act on EGCs, with their temporal effects on motility relayed through EGCs. Once rhythmic secretion by EGCs is lost, this signaling input is interrupted, and microbial metabolites can no longer drive colonic motility at the appropriate time [43]. These findings indicate that microbial metabolic rhythms not only influence colonic motility but can also act on EGCs and alter their functional state. However, direct time-resolved human evidence establishing EGC-specific circadian disruption as a driver of colonic motility desynchronization remains lacking.

### 3.4. An EGC-Mediated Vicious Cycle of Circadian Low-Grade Inflammation Perpetuates and Entrenches Gut Jet Lag

Persistent inflammatory and microbial stimulation can induce reactive changes in EGCs [70]. In primary EGCs cultured in vitro after isolation from the small-intestinal myenteric plexus of neonatal rats, Von Boyen et al. [71] found that pro-inflammatory cytokines such as TNF-α and IL-1β markedly increased both the proportion of glial fibrillary acidic protein (GFAP)-positive EGCs and GFAP expression. Inflammatory or microbial stimulation can further activate pro-inflammatory signaling pathways in EGCs, including the S100B–RAGE/NF-κB–iNOS axis, increasing the production of NO and other inflammatory mediators and thereby further amplifying local inflammatory responses [72]. Immune-cell inflammatory responses are likewise constrained by the circadian clock. Bmal1 regulates the circadian trafficking of Ly6Chi inflammatory monocytes in mice, whereas loss of clock-gene function results in sustained inflammatory responses in these cells throughout the day [73]. Immune dysregulation and IBD-like colitis have also been observed in mice with an impaired intestinal clock, whereas restoration of the intestinal clock by timed feeding markedly attenuates inflammation [74]. Together, these findings indicate that when rhythmic secretion by EGCs collapses, their pro-inflammatory programs lose circadian gating, while immune cells no longer switch their inflammatory responses across the circadian cycle, allowing low-grade inflammation to persist throughout the day. Meanwhile, the circadian pattern of EGC-derived GSNO release is lost, further aggravating temporal disruption of intestinal epithelial barrier permeability, promoting the translocation of microbial products, and amplifying local immune stimulation [28].

Taken together, EGC rhythm collapse, reactive glial activation, circadian low-grade inflammation, further suppression of the EGC molecular clock, and subsequent desynchronization of the microbiota–motility system may form a potential self-reinforcing positive-feedback loop (Figure 3). This positive-feedback cascade represents a testable hypothetical model explaining how reversible circadian disturbance could progress into persistent gut-jet-lag-associated pathology. It offers a potential pathophysiological rationale for the chronic, relapsing nature of some chronic functional constipation cases and the limited efficacy of symptomatic laxative-only management, yet direct experimental validation, particularly through time-resolved studies in humans, is still lacking.

### 3.5. Differential Molecular Features of EGC Circadian Rhythm Disruption Across Constipation Subtypes: Slow-Transit Constipation and Pelvic Floor Dyssynergia

EGC circadian rhythm disruption represents the starting point of CMM axis desynchronization, and the broader the extent of EGC rhythm disruption, the greater the degree of CMM axis desynchronization. The two most common subtypes of functional constipation provide a clear contrast: slow-transit constipation (STC) involves the entire colon, whereas pelvic floor dyssynergia (PFD) is confined to the distal outlet.

Evidence from animal models and human surgical specimens suggests that patients with STC may exhibit widespread EGC damage throughout the colon [75]. However, current evidence remains insufficient to determine whether temporal abnormalities of EGCs represent a primary cause of the disease or a secondary change arising during the progression of constipation. In a loperamide-induced animal model of STC, EGCs exhibit mitochondrial damage, increased oxidative stress, and reduced levels of glial trophic factors such as GDNF, whereas restoration of PINK1/Parkin-mediated mitophagy in EGCs improves constipation [76]. This injury initially weakens trophic support for the motility apparatus. GDNF secreted by EGCs provides important trophic support for myenteric neurons, and reduced GDNF is accompanied by widespread loss of ICC networks across all colonic segments in patients with STC [77]. Excitatory neurotransmitters in the myenteric plexus are also reduced, while neurons and EGCs undergo reactive and degenerative changes, driving the entire enteric neuroglial network toward degeneration [75,78]. The motility apparatus consequently weakens, with the frequency, amplitude, and duration of morning HAPCs all lower than those in healthy controls [79]. At the same time, the temporal regulatory capacity of EGCs is also impaired. Importantly, direct human recordings of circadian oscillations in EGC clock genes from living colonic tissue of patients with STC are still lacking. The inference of colon-wide attenuation of EGC rhythmicity in this subtype is therefore based primarily on jet-lag animal models and indirect evidence from pathological alterations in the enteric neuroglial–ICC network in patients with STC. Temporally resolved sampling of human colonic tissue will be required to further validate this hypothesis. Mediators such as GDNF are therefore not only secreted at insufficient levels but also lose their circadian rhythmicity. Reduced motility leads to retention of luminal contents and a concurrent loss of host-derived temporal signals, with consequent changes in microbial ecology and metabolism. In the fecal metagenome and metabolome of patients with delayed transit, the distributions of short-chain fatty acids, including acetate, propionate, and butyrate, as well as multiple bile acids, differ markedly from those in individuals with normal transit, and butyrate-producing Roseburia is positively correlated with butyrate levels [80]. With both trophic and temporal support disrupted, weakened motility and arrhythmic microbial metabolism converge across the entire colon, resulting in global CMM axis desynchronization and the most pronounced gut jet lag.

PFD, by contrast, shows the opposite pattern, with impairment confined to the distal outlet. Unlike STC, it is not characterized by colon-wide degenerative changes; instead, the primary abnormality lies in the coordination of defecation. Approximately one-third of patients with chronic constipation have an evacuation disorder, in which the abdominal and pelvic floor muscles fail to coordinate appropriately during defecation [81]. Such anorectal dysfunction is highly prevalent among outpatients with constipation, whereas colonic transit time may remain normal. These abnormalities do not necessarily occur in parallel, indicating that the lesion is largely confined to outlet coordination while proximal colonic transit is generally preserved [82]. Defecation depends on coordinated coupling between outlet sensation and pelvic floor relaxation: the urge to defecate must first be perceived before the pelvic floor muscles can relax appropriately during defecation. In PFD, both components are impaired. When propulsive waves driven by the morning gastrocolic reflex reach the outlet, evacuation is hindered because the pelvic floor muscles fail to relax appropriately. At the same time, reduced sensation in the distal rectum weakens the urge-to-defecate signal and consequently impairs the defecation reflex. Reduced rectal sensation has been confirmed in large-scale studies as a major feature of this subtype [83]. Single-cell studies of the human intestine further show that EGCs are not a homogeneous population but comprise glial subpopulations with distinct molecular features and spatial distributions, providing a cellular basis for selective functional abnormalities in specific intestinal regions or EGC subpopulations [84]. Taken together with current evidence on the spatial distribution of these cellular subpopulations, temporal disruption of EGCs in patients with PFD may be predominantly confined to the distal colon and rectum, whereas EGC clocks and microbial circadian rhythms in the proximal colon may remain relatively preserved. Accordingly, only localized CMM axis desynchronization may occur, resulting in a milder degree of gut jet lag. This hypothesis, however, still requires validation using temporally resolved biopsy samples from the human distal rectum.

Taken together, differences in the severity of gut jet lag across functional constipation subtypes may be explained by differences in the intestinal regions affected by EGC injury and the EGC subpopulations involved. STC is characterized by widespread injury to myenteric EGCs throughout the colon and global CMM axis desynchronization, whereas PFD is characterized by localized temporal defects of EGCs in the distal rectum and localized CMM desynchronization. Mixed constipation represents an overlap of these two patterns (Table 1). This subtype differentiation model still requires further support from human histological evidence.

## 4. Multi-Omics Evidence from Human Tissues and Preclinical Animal Models Supporting the Hypothesis of EGC Temporal Disruption

The mechanisms described above suggest that EGC rhythm abnormalities can propagate stepwise along the host clock–microbiota–colonic motility cascade and drive CMM axis desynchronization. However, this continuous pathological chain has not yet been fully validated in humans with CFC. Current evidence derives from colonic tissues of patients with constipation, animal models of circadian disruption, and populations exposed to shift work or sleep disturbances, providing support for this mechanism at multiple levels, including tissue injury, cellular clocks, microbial omics, and glial mediators.

### 4.1. Human Tissue Evidence of Gut–Glial Network Damage in Patients with Chronic Functional Constipation

The human colon exhibits a distinct molecular clock and circadian rhythm in motility. In patients with slow-transit constipation, however, the most prominent alteration is not a uniform reduction in colonic activity throughout the day, but a marked attenuation of the normal day–night difference. Camello-Almaraz et al. [85] performed continuous 24 h measurements of the human colonic muscularis and found that Bmal1, Per1, and Clock all exhibited rhythmic expression, demonstrating measurable amplitude and phase variations in the local colonic clock. Consistent with this normal temporal organization, 24 h ambulatory colonic manometry showed that patients with STC had significantly fewer colonic pressure waves and lower motor activity during the daytime, whereas nocturnal activity differed relatively little from that of healthy individuals. The normal increases in motility after morning awakening and meals were also markedly attenuated [86]. In addition, the incidence, number, and propagation of HAPCs were all significantly reduced in patients with STC compared with healthy controls. Thus, the temporal abnormality in STC is characterized primarily by attenuation of the motility peak during the active phase, while the nocturnal low-motility state is relatively preserved, resulting in an overall reduction in the amplitude of the circadian rhythm of colonic motility.

Bassotti et al. [87] compared colonic surgical specimens from 26 patients with severe refractory slow-transit constipation and 10 controls and found a marked reduction in S100-positive glial cells within the myenteric and submucosal plexuses, accompanied by decreases in enteric neurons and ICCs. These findings indicate widespread injury to the enteric neural–glial–pacemaker network in STC. Importantly, the study used full-thickness colonic surgical specimens rather than conventional endoscopic mucosal biopsies, and its endpoints were limited to cell numbers and tissue structure, without assessing temporal changes in EGC clock-gene expression. Thus, the study demonstrates that the pathological process of constipation involves the cellular network in which EGCs reside, but it cannot directly determine whether these injured EGCs also exhibit reduced molecular-clock amplitude or phase misalignment.

Recent single-cell studies of the human intestine have further shown that EGCs comprise subpopulations with distinct transcriptional profiles and spatial distributions, raising the possibility that abnormalities confined to specific intestinal regions or EGC subpopulations may be masked in tissue-averaged expression profiles [48]. Therefore, the reduced daytime and morning motility peaks and relative preservation of nocturnal activity observed in STC occur against the same pathological background as EGC–neuron–ICC network injury, suggesting that the abnormalities are more likely to manifest as attenuated local clock amplitude, reduced intercellular phase coordination, or insufficient clock-controlled functional output, rather than as a sustained reduction in the expression of all clock genes throughout the day.

### 4.2. Inherent Limitations of Human Tissue Evidence

Although colonic surgical specimens from patients with constipation have provided evidence of injury to the enteric neuroglial network, substantial limitations remain in human time-resolved tissue sampling. Nearly all available specimens are derived from surgical resections, and 24 h biopsy cohorts with strictly controlled circadian sampling times are lacking. Conventional endoscopic mucosal biopsies rarely capture the intact myenteric neural–EGC network, while cross-sectional specimens cannot readily distinguish whether EGC rhythm abnormalities represent an initiating factor in constipation or a secondary alteration that develops after disease onset. In addition, rodents are nocturnal animals whose activity–rest phases are opposite to those of humans; therefore, phase changes observed in animal experiments cannot be directly equated with circadian changes in humans.

Notable inter-species differences exist in the enteric-neuron-to-enteric-glial-cell ratio between mice and humans. The human myenteric plexus possesses substantially higher glia-to-neuron ratios compared with mouse enteric ganglia, and such ratios further differ between submucosal and myenteric plexus compartments [88]. Inflammatory stimuli, as well as circadian perturbations such as trans-meridian travel and chronic jet-lag, can trigger reactive enteric glial activation and glial–neuronal proliferative remodelling [10,71]. Such plastic glial–neuronal remodelling encompasses reactive glial activation, shifts in glial mediator profiles, and proliferative responses within enteric ganglia [32]. Activated enteric glia can alter the release of key paracrine mediators including GDNF, GSNO, pro-inflammatory cytokines and neurotrophic factors [32,41]. These shifts in mediator concentration, in turn, tune the excitability of enteric neurons, modify synaptic communication within myenteric circuits, and consequently reshape downstream colonic contractile behaviour. Because the baseline glia–neuron cellular composition differs between species, the magnitude and functional outcome of glial-neuronal remodelling observed in mouse experiments cannot be straightforwardly extrapolated to human colonic motility pathophysiology.

It should also be noted that enteric glial cells and subsets of interstitial gut cells are mechanically fragile during tissue dissociation for single-cell multi-omics workflows [89]. Technical artifacts originating from differential cell loss and variable cell recovery may introduce sampling bias [90]. Accordingly, multi-omics datasets derived from intestinal tissues require careful contextual interpretation, and observations obtained from rodent multi-omics studies cannot be uncritically generalized to human gut pathophysiology.

Taken together, current human evidence is derived primarily from surgical resection specimens and cross-sectional samples, while temporally resolved human biopsy data collected at multiple time points remain scarce. Animal models provide important mechanistic clues, but species differences limit their direct applicability to humans; therefore, these findings should be regarded as supportive evidence rather than directly extrapolated to the pathophysiology of human CFC.

### 4.3. Integrated Omics Evidence for Temporal Interactions Between EGCs and the Microbiota in Preclinical Animal Models

Animal studies with temporal profiling first revealed clear phase changes in EGCs themselves. Magaña et al. [14] found that EGCs exhibit stable, near-24 h cell-autonomous oscillations and can undergo phase resetting in response to feeding–fasting cycles. When feeding was shifted to the inactive phase, EGCs, enteric neurons, smooth muscle cells, and other intestinal wall cells exhibited phase shifts to varying degrees, whereas ICCs were relatively insensitive to feeding entrainment, thereby disrupting the pre-existing synchrony among these cell types. These findings indicate that the early effects of circadian perturbation are not simply reflected by increases or decreases in the expression of individual clock genes; rather, disruption first emerges in the temporal coordination among different cell types within the intestinal wall.

These phase alterations can subsequently extend to the microbiota and its metabolic rhythms. For example, following intestinal epithelial Bmal1 deletion, the number of microbial taxa exhibiting circadian oscillations was markedly reduced, while multiple previously rhythmic taxa, including Lachnospiraceae and Ruminococcaceae, lost or altered their oscillatory patterns; abnormalities were also observed in microbiota-derived metabolites such as branched-chain fatty acids and secondary bile acids [31]. In a simulated shift-work model, desynchronization of gastrointestinal peripheral clocks was likewise observed, accompanied by attenuated microbial rhythms and alterations in SCFA- and bile acid-related metabolic functions. Moreover, transplantation of the microbiota from these mice into germ-free recipients also altered peripheral clock genes and metabolism-related clock-controlled genes in the recipients [52].

## 5. Current Research Gaps and Future Directions

Although existing studies support the involvement of EGCs in the temporal regulation of the CMM axis at multiple levels, including the cell-autonomous clock, temporal changes in the microbiota, glial mediators, and colonic motility, a comprehensive temporal regulatory network explaining how the core molecular clock of EGCs controls their functional outputs remains to be established. Although colonic tissues from patients with constipation have revealed injury to the enteric neuroglial network, and the human colon and enteric neural network possess the molecular machinery required for core clock function, direct human evidence integrating constipation phenotypes, EGC identity, and circadian clock alterations within the same study design is still lacking [87]. Future studies could integrate EGC-specific time-resolved transcriptomics, chromatin accessibility profiling, proteomics, and secretomics, together with analyses of BMAL1/CLOCK binding sites using ChIP-seq, CUT&Tag, and related approaches. Such studies could distinguish effector molecules that merely exhibit time-dependent expression from clock-controlled targets directly regulated by the core clock, thereby establishing a continuous regulatory chain linking core clock genes, clock-controlled effector molecules, glial mediators, the microbiota, and motility, and clarifying how EGCs translate molecular timing information into functional rhythms of the CMM axis.

Furthermore, defining this molecular network alone is insufficient to establish the causal role of EGC clock abnormalities in CMM axis desynchronization. Existing models, including SCN Bmal1 deficiency, simulated shift work, mistimed feeding, and intestinal clock disruption, can all induce alterations in gastrointestinal peripheral clocks and microbial rhythms. However, because these models simultaneously affect multiple intestinal wall cell types and systemic entrainment signals, they cannot readily isolate the specific contribution of EGC clock disruption [52]. Recent studies have also revealed substantial heterogeneity among EGC subpopulations and regional differences along the intestine [32,48]. Future studies should therefore establish EGC-specific models of rhythmic disruption involving Bmal1, Clock, and Per/Cry, while simultaneously assessing EGC oscillations, circadian changes in the microbiota, SCFA and bile acid metabolic rhythms, and colonic motility. In parallel, phase-stratified human colonic single-cell time-resolved transcriptomic cohorts could be established without requiring repeated biopsies from the same individual.

The hypothesis linking EGC dysfunction, CMM axis desynchronization, and gut jet lag may have clinical implications for chronic functional constipation associated with modern lifestyles. First, in populations at high risk of constipation, such as shift workers and individuals with chronic sleep deprivation, potential circadian disruption should be considered as an underlying pathogenic background in addition to conventional laxative treatment. Second, this hypothetical framework may inspire future exploratory biomarker research; however, it does not provide validated biomarkers or ready-to-use clinical decision-making tools for patient risk stratification at present. Third, it provides a theoretical basis for identifying novel therapeutic targets directed at the EGC circadian clock. However, all of these translational concepts remain hypothetical and require substantial validation in human studies before they can be applied in clinical practice. Future work should also continue to dissect contributions from other gut cellular players within this orchestrated circadian network, not only focusing on enteric glial-dependent mechanisms.

## 6. Limitations

Current mechanistic evidence is derived predominantly from rodent models of circadian disruption, whereas EGC-specific, multi-time-point temporal data from the colons of patients with constipation remain limited. This constrains the direct extrapolation of animal findings to humans. Humans and rodents differ in activity phase, feeding patterns, hormone secretion, gastrointestinal functional rhythms, as well as neuro-glial anatomical features [3,91]; experimental daytime–nighttime time windows from rodent studies cannot be directly mapped onto human clock-time, and comparisons based on active–rest phases are therefore more appropriate [3]. Meanwhile, most existing human studies rely on sampling at a single or only a few time points, with substantial heterogeneity across studies in sampling time, definitions of circadian disruption, and methods of microbiota analysis [92]. Consequently, direct time-resolved experimental evidence for the EGC phase shifts, intercellular desynchronization, and attenuated rhythm amplitude observed in animal models remains difficult to obtain in human colonic tissues.

Current human data are likewise insufficient to determine the temporal relationship between EGC rhythm abnormalities and the disease course of CFC. Cross-sectional studies and sparse time-point sampling generally only capture the disease state at a single disease stage and cannot establish whether EGC rhythm alterations precede the onset of constipation symptoms, emerge concurrently with disease onset, or arise secondarily from prolonged motility dysfunction, dysbiosis, and local inflammation [93]. Consequently, it remains unclear whether EGC rhythm abnormalities in CFC act primarily as an initiating factor, a disease-maintaining factor, or a secondary alteration during chronic disease progression. This uncertainty also limits clinical mapping of distinct pathological stages, including early gut jet lag, complete gut jet lag, and inflammatory entrenchment.

This work is a hypothesis-generating narrative review rather than a systematic review or meta-analysis. No formal pre-registration, comprehensive systematic literature screening, or quantitative meta-analytic synthesis was performed. We synthesize published evidence to construct a testable conceptual framework instead of quantifying effect sizes. Clinical risk-stratification concepts presented herein remain purely theoretical and cannot be implemented in real-world clinical settings without sufficient human validation studies. Selective-citation bias cannot be fully excluded. While this manuscript focuses on gut circadian-biology- and enteric-glia-related literature, we acknowledge that additional valuable publications addressing other intestinal cell types also substantially advance our understanding of gut circadian physiology.

## 7. Conclusions

Chronic functional constipation (CFC) is highly prevalent in contemporary populations. Gut circadian homeostasis relies on orchestrated signaling across multiple intestinal cell populations, and circadian disturbance (gut jet lag) represents an important modifiable risk factor for CFC [8]. Within this multi-cellular context, this review proposes a hypothetical framework focusing on SCN–EGC–microbiota–colonic motility CMM-axis temporal disruption. Under physiological conditions, EGCs can serve as one class of secondary peripheral clocks within the gut multi-cell network, receive central entrainment signals, and coordinate circadian oscillations of the microbiota and the temporal functions of ICCs and PDGFRα^+^ cells, thereby maintaining multilevel temporal synchrony across the CMM axis. External circadian perturbations can attenuate molecular clock oscillations and induce phase shifts in EGCs, progressively leading to first-layer host–microbiota desynchronization (early gut jet lag) and second-layer desynchronization of the intestinal wall motility network (complete gut jet lag). EGC-mediated circadian low-grade inflammation may further establish a positive-feedback loop that entrenches an initially reversible rhythmic disturbance into a persistent pathological state. Most of the pathological inferences proposed in this review represent testable hypotheses derived from animal studies and indirect evidence from human tissue specimens and therefore require further validation in EGC-specific genetic intervention models and human time-resolved multi-omics cohorts, and should not be interpreted as proven human disease mechanisms. By delineating the major evidence gaps in this field, this review provides a theoretical basis for future basic research on gut chronobiology and on constipation-targeted strategies directed at temporal regulatory cells within the intestinal wall.

## Figures and Tables

**Figure 1:**
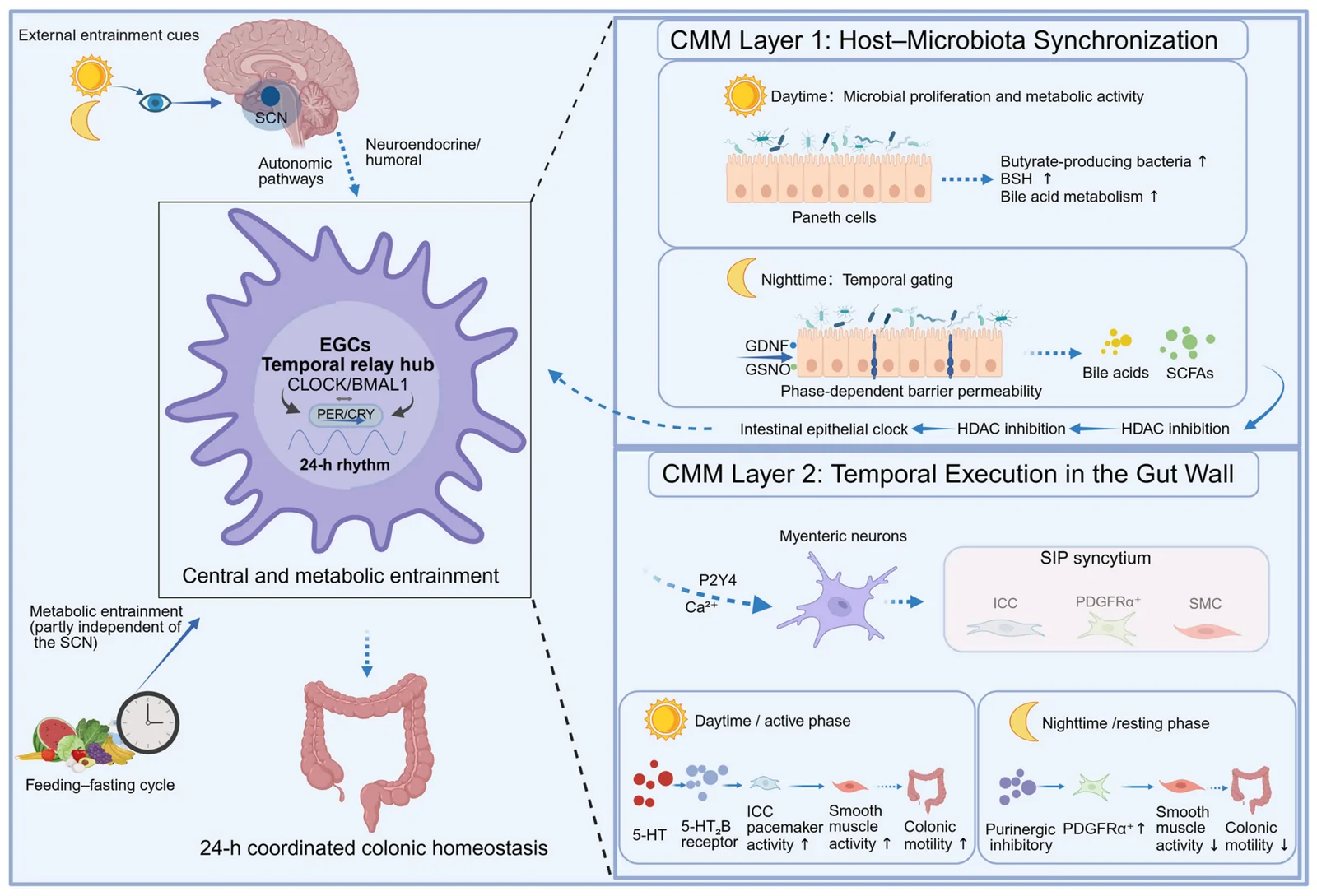
Physiological homeostasis: Schematic representation of SCN–EGC-mediated dual-layer temporal coupling within the CMM axis. External light cues entrain the suprachiasmatic nucleus, which conveys central timing information to enteric glial cells through autonomic pathways and neuroendocrine/humoral signals, whereas the feeding–fasting cycle provides an additional metabolic entrainment signal that is partly independent of the SCN. As an Interstitial temporal relay node, EGCs integrate these timing cues and coordinate two interconnected layers of the Chrono-Microbiota–Motility axis. In CMM Layer 1, EGC-mediated host–microbiota synchronization shows distinct daytime and nighttime patterns: daytime regulation supports microbial proliferation and microbial bile acid metabolism through the Paneth cell–epithelial interface, whereas nighttime EGC-derived GDNF and GSNO contribute to phase-dependent barrier permeability and temporal gating of microbial metabolite exchange. Microbiota-derived SCFAs can in turn feed back on the intestinal epithelial clock through HDAC inhibition, thereby contributing to bidirectional host–microbiota coupling. In CMM Layer 2, EGCs relay temporal information to the gut wall motor network, including myenteric neurons and the SIP syncytium composed of ICCs, PDGFRα^+^ cells, and smooth muscle cells. During the active phase, 5-HT-dependent enhancement of ICC pacemaker activity promotes colonic motility, whereas during the resting phase, PDGFRα^+^ cell-mediated purinergic inhibition contributes to reduced motility. Together, these interactions maintain coordinated 24 h colonic homeostasis. Solid arrows indicate experimentally supported interactions derived from published studies; dashed arrows denote testable hypothetical/proposed mechanistic cascades that still require direct empirical validation. Abbreviations: BMAL1, brain and muscle ARNT-like 1; BSH, bile salt hydrolase; Ca^2+^, calcium ion; CLOCK, circadian locomotor output cycles kaput; CMM, Chrono-Microbiota–Motility; CRY, cryptochrome; EGCs, enteric glial cells; GDNF, glial cell line-derived neurotrophic factor; GSNO, S-nitrosoglutathione; HDAC, histone deacetylase; ICC, interstitial cell of Cajal; PDGFRα^+^, platelet-derived growth factor receptor α-positive; PER, period; P2Y4, purinergic receptor P2Y4; SCFAs, short-chain fatty acids; SCN, suprachiasmatic nucleus; SIP, smooth muscle cell–ICC–PDGFRα^+^ cell syncytium; SMC, smooth muscle cell; 5-HT, 5-hydroxytryptamine; 5-HT_2_B, 5-hydroxytryptamine receptor 2B. Symbols: ↑, increased; ↓, decreased.

**Figure 2:**
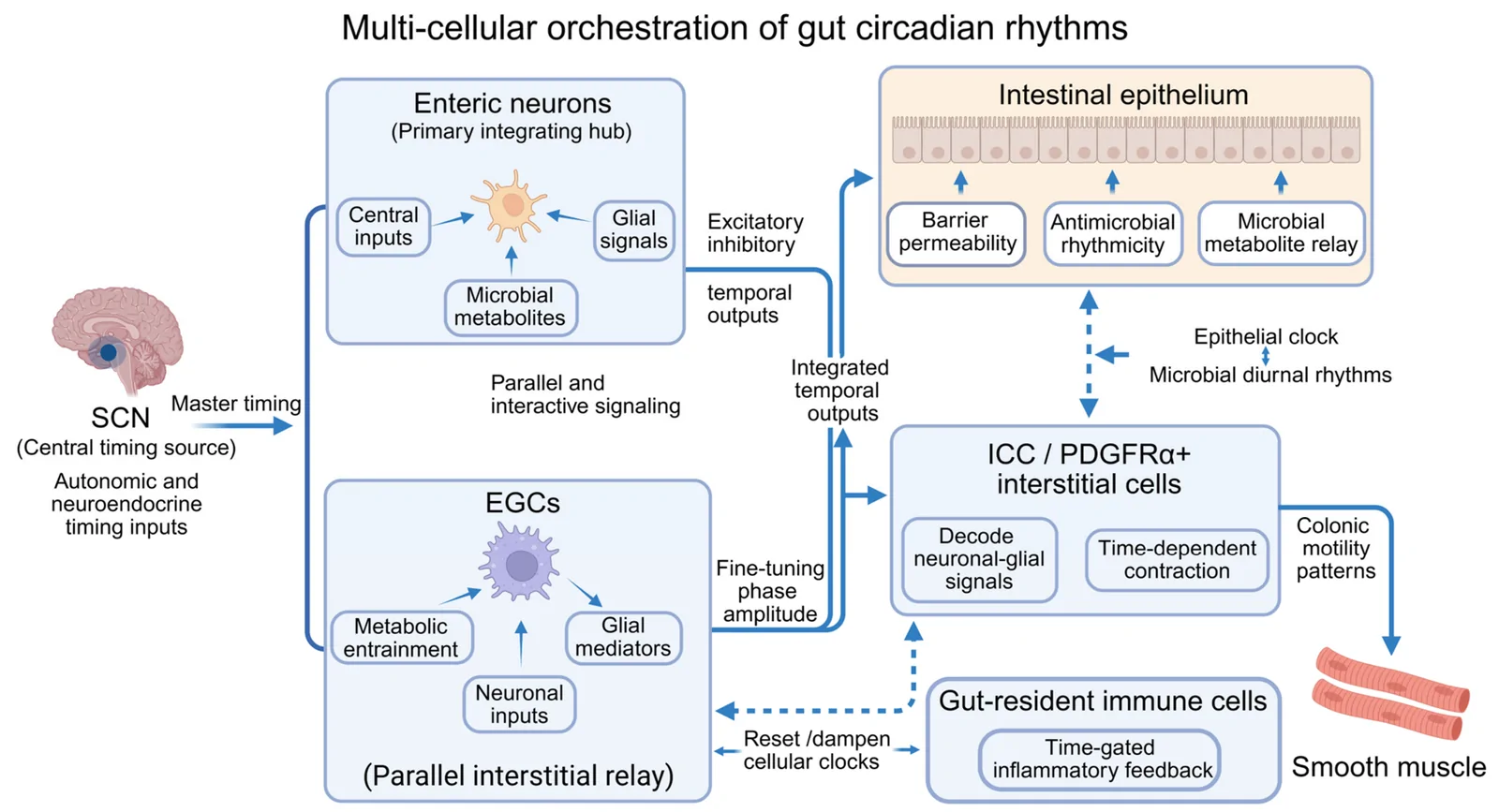
Multi-cellular orchestration of gut circadian rhythms. The gut circadian network is organized through hierarchical signal propagation and reciprocal feedback. The SCN provides central master timing, which is integrated by enteric neurons and transmitted in parallel with interstitial signals from EGCs. These coordinated inputs regulate intestinal epithelial cells and interstitial pacemaker cells, leading to time-dependent colonic motility. Epithelial–microbial interactions and immune-mediated feedback further calibrate the network. EGCs represent one parallel interstitial relay component within this multi-cellular system. In this schematic, solid arrows depict directional signaling and regulatory inputs or outputs, whereas dashed arrows highlight temporal coupling and feedback relationships. Double-headed arrows denote reciprocal interactions. The integrated temporal network represents a conceptual framework and has not been directly validated as a whole.

**Figure 3:**
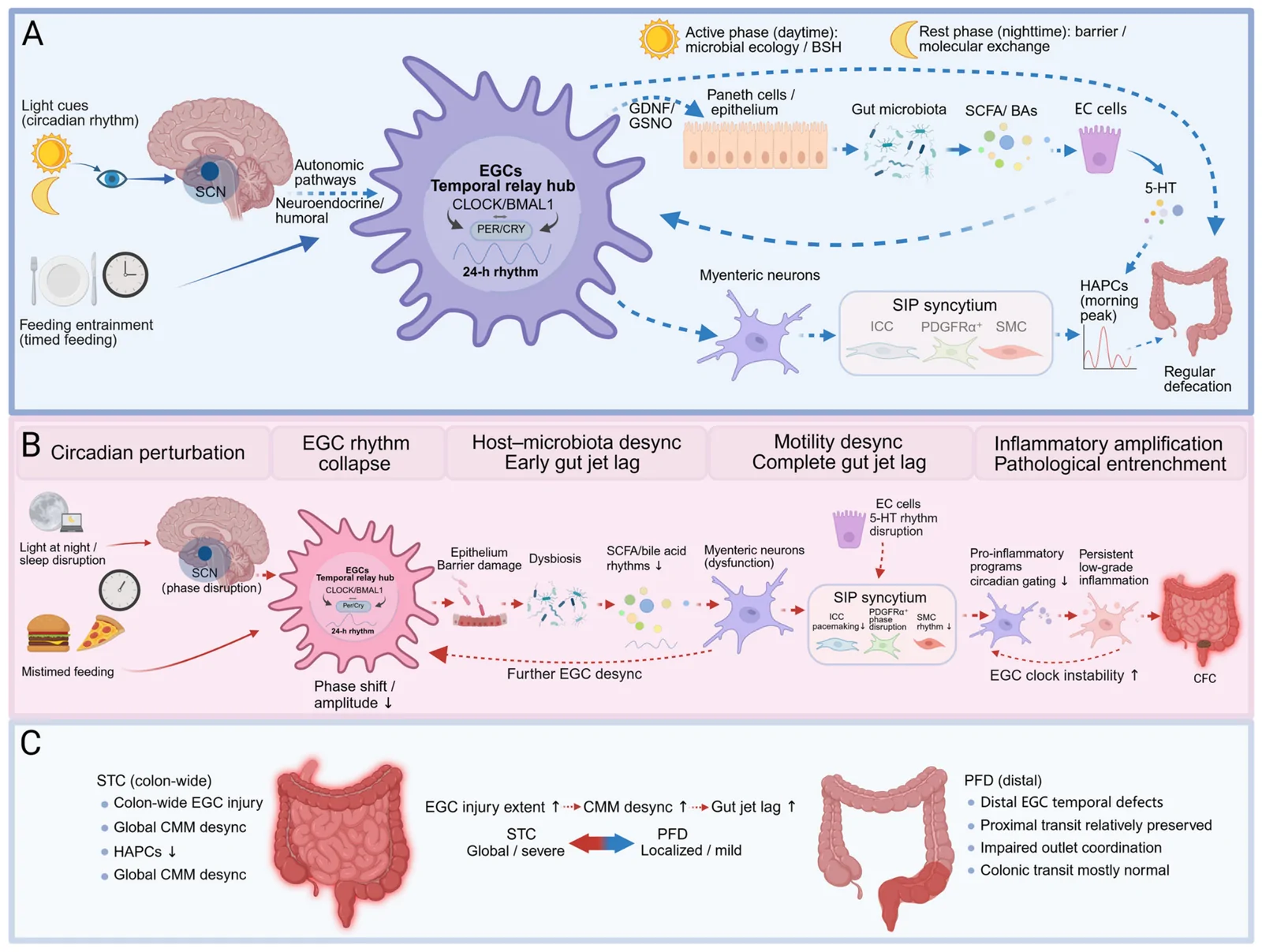
Schematic overview of SCN-EGC-centered CMM-axis temporal homeostasis and pathological desynchronization underlying gut-jet-lag-associated chronic functional constipation. (**A**): Physiological state. The suprachiasmatic nucleus conveys central timing information to enteric glial cells. As core relay nodes of the Chrono-Microbiota–Motility axis, EGCs mediate bidirectional temporal communication between gut microbiota and colonic motility effector cells (ICC, PDGFRα+ cells) to maintain multi-layered temporal synchronization. (**B**): Pathological cascade triggered by external circadian perturbation. Misaligned timing cues induce EGCs clock phase-shift and oscillation decay, initiating sequential CMM-axis desynchronization: early host–microbiota temporal decoupling, followed by full motility-network desynchronization (complete gut jet lag). EGC-driven temporal low-grade inflammation forms a self-reinforcing positive-feedback loop to lock-in pathological desynchronization. (**C**): Subtype-specific injury patterns: pan-colonic EGCs impairment in slow-transit constipation, versus distal-restricted EGCs temporal dysfunction in pelvic-floor dyssynergia. Solid arrows indicate functional interactions supported by published experimental studies; dashed arrows denote proposed temporal coupling, feedback, or mechanistic links that have not yet been directly validated. This schematic focuses on EGC-associated mechanistic branches; additional cell types within the gut circadian network contribute to these physiological and pathological processes but are not comprehensively depicted here. Abbreviations: This schematic integrates experimentally supported local functional interactions with a proposed framework of EGC-dependent temporal coupling. Direct time-resolved validation is still required for the SCN-to-EGC relay, the complete pathological cascade and circadian inflammatory feedback loop, and the proposed colon-wide versus distal patterns of EGC circadian disruption in STC and PFD. BMAL1, brain and muscle ARNT-like 1; CFC, chronic functional constipation; CLOCK, circadian locomotor output cycles kaput; CMM, Chrono-Microbiota–Motility; CRY, cryptochrome; EC, enterochromaffin cell; EGCs, enteric glial cells; HAPCs, high-amplitude propagated contractions; ICC, interstitial cell of Cajal; PDGFRα^+^, platelet-derived growth factor receptor α-positive; PER, period; PFD, pelvic floor dysfunction-related constipation; SCFA, short-chain fatty acid; SCN, suprachiasmatic nucleus; SIP, smooth muscle cell–ICC–PDGFRα^+^ cell syncytium; SMC, smooth muscle cell; STC, slow-transit constipation; 5-HT, 5-hydroxytryptamine. Symbols: ↑, increased; ↓, decreased.

**Table 1:** Comparison of EGC rhythm disruption and CMM axis desynchronization between two constipation subtypes.

Indicators	Slow-Transit Constipation (STC)	Pelvic Floor Dyssynergia (PFD)
Extent of EGC injury [75,81,84]	Widespread injury throughout the colon	Localized injury in the distal colon and rectum
Major pathological features [75,76,77,81,83]	EGC injury, reduced GDNF, and degeneration of the neural–ICC network	Impaired outlet coordination, reduced rectal sensation, and localized EGC abnormalities
EGC temporal status [40,43,84]	Widespread rhythm attenuation and desynchronization	Localized temporal defects in the distal colon and rectum
Microbial metabolism [80]	Abnormal SCFA and bile acid metabolic profiles	Limited temporal evidence
Motility characteristics [79,81,82]	Reduced HAPCs and an attenuated morning motility peak	Relatively preserved proximal colonic transit with impaired outlet evacuation
CMM axis desynchronization [8,14,43,80,82,84]	Global desynchronization (severe gut jet lag)	Localized desynchronization (mild gut jet lag)
Colonic transit [78,82]	Prolonged	Mostly normal

Footnote: All subtype-differentiated pathological patterns shown in this table represent testable hypotheses and have not been directly validated in human temporally resolved biopsy cohorts. Abbreviations: CMM, Chrono-Microbiota–Motility; EGC, enteric glial cell; GDNF, glial cell line-derived neurotrophic factor; HAPC, high-amplitude propagated contraction; ICC, interstitial cell of Cajal; PFD, pelvic floor dyssynergia; SCFA, short-chain fatty acid; STC, slow-transit constipation.

## Data Availability

No new data were created or analyzed in this study. Data sharing is not applicable to this article.

## Abbreviations

The following abbreviations are used in this manuscript:

EGCs	Enteric glial cells
CMM	Chrono-Microbiota–Motility
ICCs	Interstitial cells of Cajal
SCN	Suprachiasmatic nucleus
SCFAs	Short-chain fatty acids
PDGFRα^+^	Platelet-derived growth factor receptor α-positive
STC	Slow-transit constipation
GDNF	Glial cell line-derived neurotrophic factor
5-HT	5-Hydroxytryptamine
CFC	Chronic functional constipation
BMAL1	Brain and muscle ARNT-like 1
CLOCK	Circadian locomotor output cycles kaput
SIP	Smooth muscle cell–ICC–PDGFRα^+^ cell syncytium
HAPCs	High-amplitude propagated contractions
PFD	Pelvic floor dyssynergia
PER	Period
CRY	Cryptochrome
GSNO	S-Nitrosoglutathione
BSH	Bile salt hydrolase
HD5	Human α-defensin 5
